# Reduction in circulating vitamin D binding protein in patients with multiple sclerosis

**DOI:** 10.1186/s12883-021-02200-0

**Published:** 2021-04-20

**Authors:** Zhila Maghbooli, Abolfazl Omidifar, Tarlan Varzandi, Tayebeh Salehnezhad, Mohammad Ali Sahraian

**Affiliations:** Multiple Sclerosis Research Center, Neuroscience Institute, Tehran University of Medical Sciences, Tehran, Iran

**Keywords:** Multiple sclerosis, Vitamin D binding protein, Vitamin D, Bioavailability of vitamin D

## Abstract

**Background:**

In this study, we aimed to determine the risk association between vitamin D binding protein (VDBP) polymorphism in patients with multiple sclerosis (MS) in a MS biobank and the difference in VDBP serum levels in MS patients who were recently diagnosed.

**Method:**

The current case-control study was performed on 296 MS patients and 313 controls. Thereafter, two common missense VDBP polymorphisms, named rs7041and rs4588, were evaluated in all the participants. Serum levels of vitamin D and vitamin D binding protein were assessed in 77 MS patients who were diagnosed since one year ago and in 67 healthy people who were matched in terms of age and sex.

**Result:**

The frequency distributions of VDBP genotypes and alleles of SNP rs7041 and rs4588 were observed to be similar in both the MS and control groups (*p* > 0.05). The VDBP haplotypes, as Gc2/Gc2, Gc1/Gc1, and Gc1/Gc2, were found to be similar in the MS and control groups (*p* > 0.05).

In subgroup analysis, circulating VDBP was lower in MS patients (Ln-VDBP (μgr/ml): 3.64 ± 0.91 vs. 5.31 ± 0.77, *p* = 0.0001) even after adjusting for vitamin D levels, body mass index, and taking vitamin D supplement. There was no significant association between VDBP haplotypes and vitamin D levels in the two groups.

**Conclusion:**

The present study suggested an association between lower levels of circulating VDBP and multiple sclerosis in newly diagnosed patients. However, the VDBP causative role in the development of MS is still unclear, so it needs more studies.

**Supplementary Information:**

The online version contains supplementary material available at 10.1186/s12883-021-02200-0.

## Background

Multiple sclerosis (MS) (OMIM 126200) is a chronic neurodegenerative, inflammatory, demyelinating disease of the central nervous system (CNS), which commonly has relapsing and remitting episodes. Many efforts have been made to elucidate the molecular mechanisms of this neurodegenerative disease. Interestingly, the vast majority of all genetic risk factors identified up to now, encode some parts of the immune system. It was accepted that the brain lesions are resulted from immune cell infiltration across the blood–brain barrier (BBB) that promotes inflammation, demyelination, gliosis, and neuroaxonal degeneration, and consequently lead to the disruption of neuronal signaling [1]. The availability of reliable biomarkers could radically alter the management of MS at the critical stages of the disease’s spectrum [2].

Up to now, some studies have identified a combination of genetic and environmental factors associated with MS [3]. Recent studies have shown that circulating vitamin D_3_ concentration can be considered as an environmental risk factor, which has profound immunomodulatory effects by stimulating the production of anti-inflammatory cytokines [4]. Moreover, epidemiologic studies have shown that serum levels of vitamin D can be considered as a significant predictor of different MS phenotypes [5]. Besides, it has been argued that the reduction in the number of attacks in MS patients is significantly correlated with the increased vitamin D levels, and augment of disability in these patients was shown to have a direct correlation with the decreased vitamin D levels [6]. Although there is a strong evidence regarding the effect of vitamin D concentration in patients with MS, the exact mechanism is not fully cleared yet.

Vitamin D Binding Protein (P02774), known as Gc-globulin, is a polymorphic protein from the albumin family, which is known as the main carrier of active metabolites of vitamin D (85–90%) [4]. The vitamin D binding protein (VDBP) gene is located on chromosome 4, sublocalized to bands 4q11-q13. Moreover, VDBP contains 458 amino acids, folded into a disulfide-bonded, with a triple-domain structure that can be divided into 2 repeated, homologous domains of 186 amino acids (domains I and II) along with a shorter domain of 86 residues at the COOH terminus (domain III). Accordingly, the two binding regions have been identified within this sequence, which were as follows: a vitamin D–binding domain localized between residues 35 and 49 (domain I) and an actin-binding domain between residues 373 and 403 (domains II and III). Notably, the actin-binding site does not appear to interact with vitamin D binding significantly [7].

Because VDBP is the key determinant of vitamin D, it can efficiently affect the sustainability, biocompatibility, and biological performance of vitamin D. Additionally, VDBP can hamper the disseminated intravascular coagulation by neutralizing serum globular actin and it can also be considered as an activator of macrophages by increasing C5a-mediated macrophage chemotaxis involved in immune system activities [8, 9]. In fact, due to the above-mentioned functions, VDBP is associated with the pathophysiology of MS [8]. Several genetic and biochemical studies were previously conducted to identify the possible association of VDBP with the prevalence, progression, and period of MS disease [4, 8, 10]. The studies showed some inconsistent results in blood and cerebrospinal fluid (CSF) levels [2, 4, 11–14]. Despite these incoherent results, it has been reported that VDBP levels could be altered in patients at different stages of MS [10, 15]. Correspondingly, this suggests the importance of VDBP concerning the pathophysiology of MS, which may be used as a potential biomarker in various stages of MS.

In this study, we aimed to determine the risk association of VDBP polymorphisms in MS patients in a MS biobank, and the difference in VDBP serum levels in newly diagnosed MS patients.

## Methods

### Study design and population

This was a case-control study designed by the Multiple Sclerosis Research Center of Tehran University of Medical Sciences. The participants of this study were the MS patients referred to MS clinic of Sina hospital, which is a pioneer MS referral center in Iran. The control group’s subjects were healthy people with no histories of inflammatory, autoimmune, and neurologic disorders, and no family history of MS. All MS patients met McDonald criteria [16] for the diagnosis. Medical history, the age at the time of diagnosis of MS, and the medications were checked by the patient’s medical records, and were then confirmed by neurological examinations. This study was conducted in terms of the Declaration of Helsinki and its human experiments were approved by Ethical Committee of Neuroscience Institute of Tehran University of Medical Sciences (IR.TUMS.VCR.REC.1397.587). The written informed consent was obtained from each participant.

The VDBP polymorphisms were determined in 296 MS patients and 313 healthy people included in this study. A subgroup, including MS patients (*n* = 77) and a healthy group (*N* = 67) with the matched age and sex, were selected to measure vitamin D, albumin, and vitamin D binding protein.

### Genotyping

Whole blood was collected from the participants and then put into EDTA-containing tubes; genomic DNA was extracted using Gene ALL DNA kit (Gene ALL, Korean) in terms of the manufacturer’s protocol. We determined concentration and purity of the extracted DNA using a Nano Drop ND-1000 spectrophotometer (MPLENT). The VDBP polymorphisms (rs7041 and rs4588) in exon 11 [Asp416Glu and Thr420Lys] were determined by polymerase chain reaction (PCR) followed by performing the restriction fragment length polymorphism analysis (RFLP). Primers for VDBP gene polymorphisms were as follows: Forward; 5′-CAA GTC TTA TCA CCA TCC TG-3′ and Reverse; 5′-GC CAA GTT ACA ATA ACAC-3′.

Moreover, PCR was performed after the first denaturation for 5 min at 94 °C, followed by 35 cycles of denaturation for 45 s at 94 °C, annealing for 1 min at 60 °C, and elongation for 1 min at 72 °C, followed by a final extension step for 10 min at 72 °C.

The VDBP PCR product was digested by 2.5 U HaeIII (for rs7041) or 2.5 U StyI (for rs4588) restriction enzymes for an overnight at 37 °C (Thermo Fisher Science, Germany, Cat No. ER0151, ER0411, respectively). All the obtained samples were stained with SYBER Green (Cinnagen, Iran) and then visualized on 2% gel agarose.

The RFLP results revealed that rs7041 genotype denoted as GG (Glu/Glu) (577 and 232 bp), TT (Asp/Asp) (nondigested bond at 809), and TG (Asp/Glu) (577, 232, and 809 bp); and rs4588 genotype denoted as CC (Thr/Thr) (809 bp), AA (Lys/Lys) (584 and 225 bp), and AC (Lys/Thr) (584, 225, and 809 bp). To confirm the PCR-RFLP results, 10% of the obtained PCR samples were directly sequenced.

### Phenotype VDBP

The VDBP genotypes contributed to the following three common different VDBP variants: Gc1F (rs7041 (A) and rs4588 (G)), Gc1S (GC1S = rs7041(C) and rs4588 (G)), and Gc2 (GC2 = rs7041 (A) and rs4588 (T)); leading to 6 phenotypes (1 s/1 s, 1 s/1f, 1 s/2, 1f/1f, 1f/2, and 2/2). It is noteworthy that the combination of rs7041(C) and rs4588 (T) does not exist in humans.

### Biochemical analysis

Levels of total 25-hydroxyvitamin D3 were measured by HPLC (Agilent 1100 series, Chem Station software) using RECIPE’s HPLC complete kit. Deionized water, HPLC-grade methanol (Merck Millipore, Cat.No.1.06007) and Acetonitrile (CH3CN) (Carlo Erba, Cat.No.412372000) were used in all the performed experiments. Serum calibrators and controls (low and high levels) for 25(OH) D3 were also obtained from RECIPE. The chromatographic column was a C18, 150 × 4.6 mm, with a 5 μm particle size (Hector-M). The mobile phase was considered as 90% acetonitrile and 10% methanol in isocratic elution mode with a flow rate of 1.0 ml/min. Additionally, the detection wavelength was 264 nm.

In this study, serum levels of VDBP were determined using polyclonal ELIZA kit (CUSABIO) in terms of the manufacturer’s instructions, with intra-assay precision: CV < 8%, and inter-assay precision: CV < 10%. Moreover, serum albumin levels were determined by enzymatic colorimetric assay [Pars-Asmun kits, Iran] using an auto analyzer [Hitachi 902, Japan]. Finally, the vitamin D bioavailability was estimated based on the ratio of 25 (OH) D3/VDBP.

### Statistical analysis

All the statistical analyses were performed using SPSS 20.0 (SPSS Inc., Chicago, IL). Data were expressed as number (%) for categorical values and mean ± standard deviation for continuous variables. Data normality was analyzed by the Shapiro–Wilk test. Circulating levels of VDBP, which had no normal distribution, so a natural log transformation was applied to correct their normality distribution. Thereafter, the comparisons between the study groups were performed using the Student’s t-test and Chi-square test for continuous variables and categorical variables (control as a reference group), respectively. The Hardy-Weinberg equilibrium (HWE) and linkage disequilibrium (LD) tests of SNP were also assessed. Notably, the participants’ demographics and clinical measures were reported using descriptive statistics. The genotype and allele frequencies as well as phenotype analysis were performed by Chi-square and Fisher exact tests, respectively. A multivariable logistic regression model was also used to determine the independent association of circulating VDBP with MS. A *p*-value of less than 0.05 (2-tailed) was considered as statistically significant level.

## Results

### Baseline characteristics

The participants were recruited in the current study from March 2018 to February 2020. A total 609 subjects were enrolled in this study, of them, 296 subjects were patients with MS (79.1% Female) with the mean age of 33.06 ± 8.96 (years old) as well as 313 control subjects (58.8% Female) with the mean age of 35.05 ± 8.41(years old). The baseline and clinical characteristics of the included participants are summarized in Table 1.

**Table 1:** Demographic and clinical characteristics of multiple sclerosis (MS) and healthy groups

Demographic characteristic	MS (*N* = 296)	Control (*N* = 313)
Age (years)†	33.06 ± 8.96	35.05 ± 8.41
Sex (Women)‡	79.1% (234)	58.8% (184)
BMI (kg/m^2^)†	24.24 ± 4.36	25.61 ± 4.76
**Clinical outcomes**		
Duration of disease (years)†	1 (5)	–
MS diagnosis age (years)†	29.52 ± 9.55	–
MS type		–
RRMS	90.2% (267)	
PPMS	3.7% (11)	
SPMS	4.4% (13)	
CIS	1.4% (4)	
RIS	0.3% (1)	

*Abbreviations*: *BMI* body mass index, *RRMS* Relapsing-Remitting Multiple Sclerosis, *PPMS* Primary-Progressive Multiple Sclerosis, Secondary-Progressive Multiple Sclerosis, *CIS* Clinically isolated syndrome, *RIS* Radiologically isolated syndrome

^†^mean ± SD, ^††^ median (IQR), ^‡%^(*N*)

### Polymorphisms in the vitamin D-binding protein gene

In our population, the minor-allele frequency was 0.48 for rs7041 (T allele) and 0.30 for rs4588 (A allele). The distribution of the studied genotypes is shown in Table 2. The observed allele frequencies were found to be consistent with Hardy-Weinberg equilibrium in all the subjects (*p* = 0.86 for rs4588, *p* = 0.74 for rs7041). In addition, genotypes in both codons were in Hardy-Weinberg equilibrium in the MS and control groups.

**Table 2:** Genotype, allele frequency and phenotype of Vitamin D-Binding Protein Gene in MS and control groups

	MS (*N* = 296)	Control (*N* = 313)	*p*-value
Genotype- rs4588			
**CC** (Thr/Thr)	48.6% (144)	50.2% (157)	0.88
AA (Lys/Lys)	8.8% (26)	9.3% (29)	
AC (Lys/Thr)	42.6% (126)	40.6% (127)	
Genotype- rs7041			
**TT** (Asp/Asp)	20.9% (62)	25.6% (80)	0.36
GG (Glu/Glu)	29.1% (86)	25.9% (81)	
TG (Asp/Glu)	50.0% (148)	48.6% (152)	
allele Freq. rs4588			
**C**	0.6993	0.7044	0.84
A	0.3006	0.2955	
allele Freq.rs7041			
**T**	0.4594	0.4984	0.17
G	0.5405	0.5015	
GC phenotype			
Gc1f, Gc1f	4.1% (12)	5.8% (18)	0.65
Gc1s, Gc1s	28.4% (84)	24.6% (77)	
Gc2, Gc2	8.1% (24)	8.0% (25)	
GC1f,GC1s	17.9% (53)	20.1% (63)	
GC1f,GC2	10.1% (30)	12.8% (40)	
GC1s,GC2	30.7% (91)	27.5% (86)	
Rare	0.7% (2)	1.3% (4)	

Genotyping was performed for rs4588 and rs7041 of VDBP gene in 295 MS patients and 313 control subjects. The Table 2 presents the genotype, allele, and phenotype frequency in the MS and control groups (the reference group is healthy people).

The dominant genotype of SNP rs4588 was CC in the two groups, and the dominant genotype of SNP rs7041 was recognized as the heterozygous TG within each one of the studied group. There was no significant difference in genotype frequencies of SNP rs7041 and rs4588 between the two studied groups (*p* > 0.05). Accordingly, the minor-allele in both groups was A for rs4588 and T for rs7041. Moreover, no significant difference was found in the minor-allele frequencies of rs4588 and rs7041 in the MS patients compared to the controls. Six possible haplotypes defined by these 2 variants are shown in Table 2; as 1 s/1 s, 1 s/1f, 1 s/2, 1f/1f, 1f/2, and 2/2. The data analysis showed that Gc2-Gc2 phenotype was similar in both groups (8.1% in the MS patients, and 8.0% in the control subjects).

### Serum levels of vitamin D, vitamin D binding protein and bioavailability of vitamin D

The serum levels VDBP were measured in the sub-samples of both groups matched in terms of age and sex; 77 MS patients and 67 control subjects (Table 3). The MS patients were diagnosed for equal to or less than one year.

**Table 3:** Demographic characteristic and biochemical analysis in MS and control sub-groups

Demographic characteristic	MS (*N* = 77)	Control (*N* = 67)	*p*-value
Age (years)†	33.68 ± 8.16	34.71 ± 6.45	0.89
Sex (Women)‡	76.9% (60)	76.6% (52)	0.94
BMI (kg/m^2^)†	24.66 ± 4.47	24.19 ± 3.81	0.49
**Clinical outcomes**			
MS Duration (month)††	4.02 (8)	–	
MS diagnosis age (years)†	33.60 ± 7.81	–	
Smoking‡	10.0% (7)	6.0% (4)	0.52
Vitamin D levels†	33.60 ± 14.91	21.55 ± 12.49	0.0001
Ln. VDBP levels (μgr/ml) †	3.64 ± 0.91	5.31 ± 0.77	0.0001
Albumin (g/dl)†	4.07 ± 0.81	4.10 ± 0.85	0.89
Taking vitamin D supplement ‡			
Regular	54.5% (42)	18.0% (12)	0.0001
Irregular	17.0% (13)	35.8%(24)	
Never	28.5% (22)	46.2% (31)	
Taking drug at the sampling time‡			
No treatment	32.5% (25)	–	
Rituximab	11.7% (9)	–	
Natalizumab	2.6%(2)	–	
Interferon B1a	26.0% (20)	–	
Glatiramer acetate	9.1% (7)	–	
Dimethyl fumarate	2.6% (2)	–	
4 Aminopyridin	1.3% (1)	–	
Fingolimod	13.0% (10)	–	
Teriflunomide	1.3% (1)	–	
Corticosteroids	–	–	

*Abbreviations*: *BMI* body mass index, *Ln* natural logarithm

^†^mean ± SD, ^††^ median (IQR), ^‡%^(*N*)

None of the patients used steroidal drugs (prednisolone, dexamethasone, and other corticosteroids) within the last 3 months. Among the studied patients, 66.2% (51 out of 77) were consuming MS medications. All the patients were RRMS and there were no comorbidities with other diseases.

The serum levels of vitamin D (ng/ml) were observed to be higher in MS patients (33.60 ± 14.91 vs. 21.55 ± 12.49, *p*-value = 0.0001) because of the regular consumption of vitamin D supplementation as a part of their MS treatment (54.5% vs. 18.0%).

The serum levels of VDBP (μgr/ml) were significantly lower in the MS patients (Ln- VDBP: 3.64 ± 0.91 vs. 5.31 ± 0.77, *p* = 0.0001), even after adjusting confounding factors, including age, sex, vitamin D levels, season of sampling (winter), and BMI (Beta = − 2.07, 95% CI, lower-upper: 0.12, 0.02–0.71, *p* = 0.02). Notably, no significant correlation was found between VDBP and vitamin D levels in these two groups (MS group: r = 0.01, *p* = 0.91, control group: r = − 0.17, *p* = 0.14).

In the MS group, there was no correlation between circulating VDBP and duration of diseases (r = − 0.07, *p* = 0.53). Correspondingly, The duration of the disease was categorized based on 3 period of 0–3, 4–6, and 7–12 months. Furthermore, no significant differences were observed among sub-classifications (Ln-VDBP (μgr/ml): 3.93 ± 1.16, 3.57 ± 0.90, and 3.48 ± 0.71, respectively, *p* = 0.22). Circulating VDBP was not significantly different between MS patients taking MS medications and those without it (Ln-VDBP (μgr/ml): 3.52 ± 0.84, vs. 3.84 ± 1.03, respectively, *p* = 0.15) even after adjusting for the factors of age, sex and duration of the disease (*p* = 0.72).

Vitamin D_3_/VDBP ratios differed between the two groups, as it had higher levels in the MS patients (mean, 95% CI lower-upper: 1.20, 0.96–1.44) compared to the control group (mean, 95% CI lower-upper: 0.13, 0.10–0.17).

### GC phenotypes and serum levels of vitamin D

To consider the association between vitamin D levels and GC isoforms, the obtained data were sub-classified based on GC2 (GC2-GC2 or GC2-GC1) isoform, 41 out of 77 in the MS sub-group carried GC2 compared with 37 out of 67 in the control’ sub-group (*p* = 0.52). In the control group, there was no significant difference in levels of 25(OH) D3 (ng/ml) between carriers GC2 and those of non-carriers (23.98 ± 13.16, 20.22 ± 11.40, *p* = 0.26). However, in the MS sub-group, the trend of serum vitamin D levels was observed to be lower in carriers GC2, but it was not statistically significant (32.99 ± 11.75, vs. 35.60 ± 17.09, *p* = 0.44). A similar result was found in total study population (Table S1).

## Discussion

To consider the possible role of VDBP in MS etiopathogenesis, we determined serum VDBP concentrations and its common polymorphisms in MS patients. In this line of research, only few studies have previously assessed VDBP levels in MS patients, which were reviewed by Gauzzi [4]. Our data revealed that circulating VDBP levels were lower in MS patients compared to the healthy group, which was independent of vitamin D levels. In contrast to our results, previous studies in this field reported no significant differences in circulating VDBP in MS patients [17–19]. On the other hand, Rinaldi et al. in their study found higher serum levels of VDBP in MS patients [9]. Nevertheless, proteomic studies performed on the discovery of CSF biomarkers in MS patients have shown lower levels of VDBP in them [20, 21]. Of note, although there is no clear reason to explain this discrepancy, it can be partly explained by the duration and course of the disease, as well as the medications used and vitamin D statuses. In the present study, circulating VDBP was assessed in newly diagnosed MS patients who were for equal to or less than one year in the remission phase and 66.2% of them were consuming medications. No evidence still exists on how the available MS medications affect VDBP levels.

Nevertheless, the alteration of its circulating levels may affect MS pathophysiology in different ways, one of which is by modifying vitamin D bioavailability.

One of the main function of VDBP is its ability to bind to the principal vitamin D metabolites: 25-hydroxyvitamin D (25(OH)D; calcidiol), as the major circulating metabolite, and 1,25-dihydroxyvitamin D (1,25(OH)2D; calcitriol), as the most active metabolite of the vitamin [22]. In serum levels of non-pregnant healthy individuals, around 85% of circulating 25(OH) D and 1, 25(OH) 2D carries via binding to VDBP with high affinity as approximately 15% are connected to albumin and less than 0.4% of them are free [23].

As expected earlier, lower VDBP concentrations led to lower VDBP-bound 25(OH) D levels and higher vitamin D bioavailability among the MS patients. As a part of the treatment strategy, vitamin D supplementation is usually recommended for MS patients. Accordingly, in the data obtained in this study, most MS patients (54.5%) used regular vitamin D supplementation (50,000 IU, per two weeks) and circulating 25OHD levels were higher in them compared to the control group. Therefore, taking vitamin D supplementation and lower VDBP levels in MS patients may be related to the increased level of free vitamin D.

The previous findings postulated that free vitamin D, which is unbound, can pass through CNS in healthy subjects [24]. One possible mechanism in this regard is based on the free hormone hypothesis by which unbound 25(OH) D and 1, 25(OH) 2D serum levels reach CNS by passing the blood brain barrier. Based on this opinion, VDBP-bound 25(OH) D is considered as a vital systemic reservoir in blood stream that is not required for entering active metabolites of vitamin D into cells [25].

Moreover, some recent studies have shown that VDBP-bound 25(OH) D is principally taken up through megalin protein acting as a receptor-mediated endocytosis in kidney [26]. It has been argued that Vitamin D-VDBP complex can also be internalized by megalin-dependent transport to pass through the blood-CSF barrier [27]. So, it can be explained that the impaired serum levels of VDBP in MS patients limit vitamin D metabolite availability in the CNS.

It was appeared that vitamin D supplementation with lower levels of VDBP in MS patients could have some beneficial effects on the disease’s activity or disability [28]. On the other hand, only 5% of circulating VDBP is carrier of vitamin D metabolites to target cells and the majority of VDBPs are consumed for their other biological functions, especially under inflammatory condition.

VDBP is known as a precursor of macrophage activation [29], presented on cell surface of monocytes [30] to mediate their effects on regulating immune responses or promoting CNS repair. As an immunomodulatory response, the activated infiltrated monocyte-derived macrophages as well as the presence of foamy macrophages in CNS can be harmful for MS patients [10] via changing CNS homeostasis [31].

Otherwise, monocyte-derived macrophages can also promote CNS repair by interacting with scavenger receptors as well as the clearance of myelin debris [32]. Some experimental studies have previously shown that monocyte-derived macrophages can synthesis trophic and anti-inflammatory factors to help the maintenance and regeneration of neurons [33]. Correspondingly, this phenomenon appears to explain the reduced VDBP concentration at early stages of MS disease that negatively affects self-protective mechanism, in order to eliminate abnormalities and also to recover a steady state of demyelination.

The other mechanism explaining the association of lower VDBP concentration and MS risk is the capacity of this protein to bind to the high affinity globular actin (G-actin) that is indispensable in actin-scavenger system. During tissue’s injury such as myelin and axonal damages, intracellular molecules release into CSF. These molecules differently act as” alarmins”, to make signal for immune system for recruiting the damaged site [34]. During tissue’s damage, actin is released into extracellular fluids, which can act as the clearance of the damaged cells and VDBP, as the main partner of G-actin, plays a fundamental role in the scavenger function. It was reported that actin levels in CSF increase in MS patients. Accordingly, these are more elevated in progressive MS patients with a trend to higher EDSS scores [35].

Based on this evidence, lower levels of circulating VDBP in MS patients could suggest impairment in danger signal of ongoing nerve damages in MS. On the other hand, it may have a protective mechanism at early stages to maintain CNS homeostasis.

In this study, we have also investigated the two common missense VDBP polymorphisms, named rs7041 and rs8455, which are considered as determinant circulating vitamin D metabolites [11]. In the current study, we have also observed no statistically significant difference in the distribution of two SNPs of VDBP and risk of MS, which is consistent with the findings of some previous studies [7, 36–39]. In a limited number of genetic studies, the association of VDBP polymorphisms with 25OHD levels has been evaluated in MS patients [40]. In a cross-sectional study performed by Laursen et al. on 1497 MS patients, it was reported that MS patients who carry rs7041 have lower circulating vitamin D levels [41]. However, we have found no significant association among rs7041, rs8455, and vitamin D levels in the studied groups, which is consistent with the findings of the Agnello et al.’s study [40].

In this study, we have also considered the combinations of these two SNPs, and Gc1F, Gc1S, and Gc2, which differ by amino acid substitutions as well as polysaccharide structures [42]. These three variants result in six common VDBP phenotypes (named GC1s/1 s, GC1s/1f, GC1s/2, GC1f/1f, GC1f/2, and GC2/2) that affect the affinity of VDBP for vitamin D ligands. Moreover, it was reported that Gc1f-1f has the highest binding affinity and Gc2–2 has the lowest one for 25(OH) D [43]. In this study, we observed similar frequency of the carriers of GC2 isoform in both of the studied groups. Of note, vitamin D levels were similar in different GC phenotypes (GC2/GC2, GC1/GC2 and GC1/GC1).

Recently, in a general population, two GWASs have identified that individuals carrying GC2 isoform have lower concentration of vitamin D in their blood [39, 44]. However, in consistent with our result, Agnello et al. observed no statistically significant different levels of vitamin D in MS patients carrying GC2 isotype [40].

The present study has found some ways for enhancing the understanding on VDBP roles in MS pathogenesis. This study has revealed different circulating levels of VDBP in newly diagnosed MS patients. We have measured 25(OH) D3 levels using HPLC method with high specificity and sensitivity. It was detected that the result of HPLC method had the highest correlation (r = 0.96) with LC-MS/MS methods [45]. Indeed, serum concentrations of VDBP was measured using polyclonal ELISA method in this study, which was not biased by VDBP genotype [39] and was compatible with liquid chromatography-tandem mass spectrometry (LC-MS) [46].

However, our study had some limitations. The design of our study was case-control that could not show the causality or protective role of VDBP in the pathogenesis of MS. To consider the pathogenesis role of VDBP, more longitudinal studies on MS patients are needed, especially at early stage of the disease. However, the results of this study can be beneficial to further identifying the role of VDBP in MS patients.

## Conclusion

In conclusion, we identified no risk association between common genetic variants of VDBP and MS. The present study revealed an independent association between lower serum levels of VDBP and the risk of MS. It still remains debatable if variation of circulating VDBP can be considered as a defense mechanism at early stages of MS to maintain CNS homeostasis or as a biomarker of MS risk. Further works are needed to establish whether VDBP could be known as a biomarker at early stages of MS.

## Supplementary Information

**Additional file 1: Table S1.** GC phenotypes and serum levels of vitamin D.

## Data Availability

The data that support the findings of this study are available from “Multiple Sclerosis Research Center of Tehran University of Medical Sciences” but restrictions apply to the availability of these data, which were used under license for the current study, and so are not publicly available. Data are however available from the authors upon reasonable request and with permission of research deputy of Multiple Sclerosis research center of Tehran University of Medical Sciences.
