# Supplementary material for: Reduction in circulating vitamin D binding protein in patients with multiple sclerosis
Source: BMC Neurol. 2021 Apr 20;21:168. doi: 10.1186/s12883-021-02200-0 (PMC8056586; doi:10.1186/s12883-021-02200-0)
Supplement: Supplementary file 1 — Additional file 1: Table S1. GC phenotypes and serum levels of vitamin D. [file 12883_2021_2200_MOESM1_ESM.docx]

**Table S1: GC phenotypes and serum levels of vitamin D:**

| **Sqrt-25(OH)D3 (ng/ml)** | | | | | |
| --- | --- | --- | --- | --- | --- |
| **GC-phenotypes** | | | |  | |
|  | Gc1/Gc1 | Gc2/Gc2 | Gc2/Gc1 | P-value | P-value after adjusting |
| MS (248) | 5.20±1.95 | 4.61±1.91 | 4.89±1.76 | 0.28 | 0.49 |
| Control (254) | 4.12±2.16 | 4.04±1.92 | 3.98±1.76 | 0.86 | 0.56 |
| Total (502) | 4.65±2.12 | 4.30±1.91 | 4.44±1.83 | 0.37 | 0.13 |

In total study population, the serum levels of vitamin D were higher in MS patients than control group (Sqrt-25(OH)D3 (ng/ml): 5.01±1.87, vs. 4.09±2.02, p=0.0001) because of taking vitamin D supplement (65.0% vs. 25.9%). There were not any significant differences in serum levels of vitamin D among VDBP phenotypes even after adjusting confounding factors (age, sex, BMI, sampling season, taking vitamin D) by univariate analysis.
